# Cytokines Induce Monkey Neural Stem Cell Differentiation through Notch Signaling

**DOI:** 10.1155/2020/1308526

**Published:** 2020-05-13

**Authors:** Min Wang, Liming Yu, Lu-Ying Zhu, Hua He, Jie Ren, Jie Pan, Xiaoyun Xie, Chunhui Cai, Lixia Lu, Haibin Tian, Li Chen, Ying Zhang, Yuehua Liu, Ce Zhang, Zhengliang Gao, Xin-Xin Han

**Affiliations:** ^1^National Key Disciplines, Key Laboratory for Cellular Physiology of Ministry of Education, Department of Neurobiology, Shanxi Medical University, Taiyuan 030001, China; ^2^College of Medicine, Jiaxing University, Jiaxing 314001, China; ^3^Oral Biomedical Engineering Laboratory, Shanghai Stomatological Hospital, Fudan University, Shanghai 200001, China; ^4^Department of Neurosurgery, Third Affiliated Hospital of Second Military Medical University, Shanghai 200438, China; ^5^Shanghai Tenth People's Hospital, Tongji University School of Medicine, Tongji University Advanced Institute of Translational Medicine, Shanghai 200092, China; ^6^Shanghai Tenth People's Hospital, Tongji University School of Medicine and Lifeng Institute of Regenerative Medicine, Tongji University, Shanghai 200092, China

## Abstract

The mammalian central nervous system (CNS) has a limited ability to renew the damaged cells after a brain or spinal cord injury whether it is nonhuman primates like monkeys or humans. Transplantation of neural stem cells (NSCs) is a potential therapy for CNS injuries due to their pluripotency and differentiation abilities. Cytokines play an important role in CNS development and repair of CNS injuries. However, the detailed cytokine signaling response in monkey neural stem cells is rarely studied. In our previous research, we isolated NSCs from the adult monkey brain and found the effects of cytokines on monkey NSCs. Now, we further analyzed the regulation mechanisms of cytokines to the proliferation of monkey NSCs such as bone morphogenic protein 4 (BMP4), BMP4/leukaemia inhibitory factor (LIF), or retinoic acid (RA)/Forskolin. The data showed that BMP4 inhibited cell proliferation to arrest, but it did not affect the stemness of NSCs. BMP4/LIF promoted the astrocyte-like differentiation of monkey NSCs, and RA/forskolin induced the neuronal differentiation of monkey NSCs. BMP4/LIF and RA/forskolin induced monkey NSC differentiation by regulating Notch signaling. These results provide some theoretical evidence for NSC therapy to brain or spinal cord injury in regenerative medicine.

## 1. Introduction

The mammalian central nervous system (CNS) has a limited ability to replace cells that are lost after injury. Transplantation of neural stem cells (NSCs) is a potential therapy for CNS injuries due to their pluripotency and differentiation abilities. NSCs differentiate into neurons, astrocytes, and oligodendrocytes, depending on complex signals. Although NSCs display the potential for neuronal differentiation *in vitro*, most of the cells differentiate into astrocyte-like cells after being transplanted *in vivo* [1, 2]. The discovery of the mechanisms by which external stimuli, like cytokines, regulate cell differentiation and signaling pathways regulate the final cell fate will be important and will benefit the nervous system repair.

Monkey NSC differentiation is controlled not only by endogenous genes, such as Notch, Wnt, and bone morphogenic protein (BMP) gene families, but also by exogenous factors such as epidermal growth factor (EGF), fibroblast growth factor 2 (FGF2), and leukaemia inhibitory factor (LIF). BMP, a member of the transforming growth factor-*β* superfamily, has been implicated as an antineural factor that restrains the proliferation and promotes the differentiation of embryonic stem cells (ESCs) into nonneural fates both *in vitro* and *in vivo* [3–5]. Moreover, BMP signaling cooperates with other developmental pathways, such as the Notch [6, 7] and Wnt signaling pathways [4, 8], to coordinate cell fate, cell proliferation, and differentiation.

LIF is a member of the interleukin-6 cytokine family. *In vivo* studies show that deletions of LIF or Stat3 disrupt the astrocyte differentiation of NSCs [9, 10], indicating that LIF may affect NSC differentiation in the developing brain by activating the transcription factor Stat3. Thus, the combination of BMP and LIF maintains the self-renewal of mouse ESCs [11]. However, few reports have examined the effect of BMP/LIF on monkey NSCs.

Retinoic acid (RA), an active derivative of vitamin A, has frequently been reported to play an important role in embryonic development, cell proliferation, and differentiation. Based on the results of *in vitro* studies, RA promotes growth arrest [12] and induces the differentiation of murine ESCs by disrupting LIF/Stat3 signaling [1, 13] or inducing the expression of different genes, like cAMP response element-binding protein (CREB) and glycogen synthase kinase 3*β* (Gsk3*β*) [10]. Therefore, RA is widely used in stem cell-based therapy and regenerative medicine to drive ESCs to differentiate into specific cell lineages [1, 14]. Meanwhile, Forskolin, an adenylate cyclase activator, potently induces the neural differentiation of adipose stem cells [15] and malignant glioma cells [16]. A high concentration of forskolin can induce ESCs to neuronal differentiation [17]. However, the signaling pathway by which RA/forskolin induces monkey NSC differentiation is unclear.

NSCs are proposed as a candidate for cellular replacement therapy due to their plasticity. The effects of various growth and differentiation factors on cell proliferation differ between cell types and species. The functions of factors like BMP4/LIF and RA/forskolin are unclear. Moreover, the signaling pathways and mechanisms that decide the fate of monkey NSCs are poorly understand. Our group has obtained NSCs from the adult monkey brain [18]. In the present study, BMP4, coupled with a low concentration of FGF2, decreased the proliferation of monkey NSCs. Monkey NSCs still maintained Sox2 expression after BMP4 treatment. BMP4/LIF or RA/forskolin treatment also decreased NSC growth and might direct monkey NSCs to differentiate into the astrocyte or neural lineage. Additionally, the Notch signaling pathway was involved in the inhibition of proliferation and induction of differentiation. We explored cytokine functions and the activation of signaling pathways during monkey NSC differentiation, and these findings may benefit researchers aiming to repair the nervous system by NSC transplantation. We also provide a possible theoretical basis for NSC-mediated cell therapy in regenerative medicine.

## 2. Materials and Methods

### 2.1. Cell Culture

A male monkey (*Macaca fascicularis*, 4 kg, 3 years old) was used to isolate the monkey NSCs, as described in our previous paper. The study strictly complied with the requirements of the Animal Welfare Act. The monkey underwent surgery under general anaesthesia in accordance with the protocol approved by the Animal Ethics Committee. The animal was euthanized after the study. We produced the monkey NSCs examined in this research using the method described in our published paper [18]. NSCs were cultured at 37°C in a 5% CO_2_ incubator with DMEM/F12 supplemented with N2, B27, GlutaMAX, penicillin/streptomycin, 20 ng/mL FGF2, 20 ng/mL EGF, and 0.5 ng/mL heparin. The monkey NSCs were digested and passaged every 6 days. HCN cells (NSCs) were derived from adult rat hippocampus SGZ (subgranular zone). NSCs had been a stable established cell line, as a gift from Dr. Fred H. Gage, Salk Institute, USA.

### 2.2. Cytokine Treatments

NSCs were plated in 6-well or 24-well plates coated with poly-L-ornithine (0.5 *μ*g/mL) and laminin (5 *μ*g/mL). The induction culture conditions are as follows: (1) control group: basal medium (DMEM/F12 containing N2, B27, and 5 ng/mL FGF2); (2) BMP4 group: basal medium supplemented with 100 ng/mL BMP4; (3) BMP4/LIF group: basal medium supplemented with 200 ng/mL BMP4 and 50 ng/mL LIF; (4) RA/forskolin group: basal medium supplemented with 1 *μ*M RA and 5 *μ*M Forskolin.

### 2.3. Immunofluorescence Staining

Cells were fixed with 4% PFA for 15 min at room temperature followed by permeabilization in PBS containing 2.5% Triton X-100 for 15 min. Then, nonspecific reactions were blocked with 3% normal donkey serum (Jackson Immuno Research) for 1 h at room temperature. Cells were incubated with the primary antibodies Sox2 (1 : 1000, R&D), Ki67 (1 : 750, Thermo Fisher), Tuj1 (1 : 300, Abcam), and S100*β* (1 : 300, Abcam) overnight at 4°C. After washing three times with 0.1% Tween-20 in PBS, cells were exposed to secondary antibodies at room temperature for 3 hours (1 : 1000, Jackson Immuno Research). Primary and secondary antibodies were dissolved in PBS supplemented with 3% BSA and 0.1% Tween-20. For nuclear staining, cells were treated by 10 ng/mL DAPI for 10 min. Cell images of the immunofluorescence staining were captured using an inverted fluorescence microscope (Nikon TE2000). A total of 6 fields of each well (3 wells/group) were randomly selected for a blinded assessment of cell number using a 10x light microscope.

### 2.4. Clone Formation Assay

Monkey NSCs were digested with dispase II (Roche) and dissociated into a single-cell suspension. After plating into 6-well plates in the growth medium, they were monitored under a microscope every 3–4 days for a total of 21 days. The medium was refreshed every 3 days. Colonies were stained with Giemsa after 21 days.

### 2.5. Cell Proliferation Assay

Monkey NSCs (5 × 10^4^ cells/well) were seeded into 96-well plates, and the MTT assay was performed to detect cell proliferation by measuring the optical density at 450 nm 0, 48, 80, and 168 h after cytokine treatments.

### 2.6. Quantitative Real-Time PCR (qPCR)

Total RNA was extracted from monkey NSCs using Trizol, according to the manufacturer's protocol. The cDNA templates were synthesized using a cDNA synthesis kit. qPCR was performed using the Real-Time PCR Reagent kit with SYBR Green dye and the reference dye ROX on an ABI 7500 Real-Time PCR System. The reaction parameters were established according to the manufacturer's instructions (40 cycles of 15 min at 95°C, 10 s at 95°C, and 32 s at 60°C). GAPDH served as an internal reference control. The primer sequences for genes are shown in Supplementary Table S1.

### 2.7. Statistical Analysis

Data are presented as the means ±standard deviations of three independent experiments and were analyzed by two-tailed Student's *t*-test. Statistical significance was set at *P* < 0.05.

## 3. Results

### 3.1. BMP4 Inhibited Monkey NSC Proliferation and Monkey NSCs Maintained Sox2 Expression

**Figure 1:**
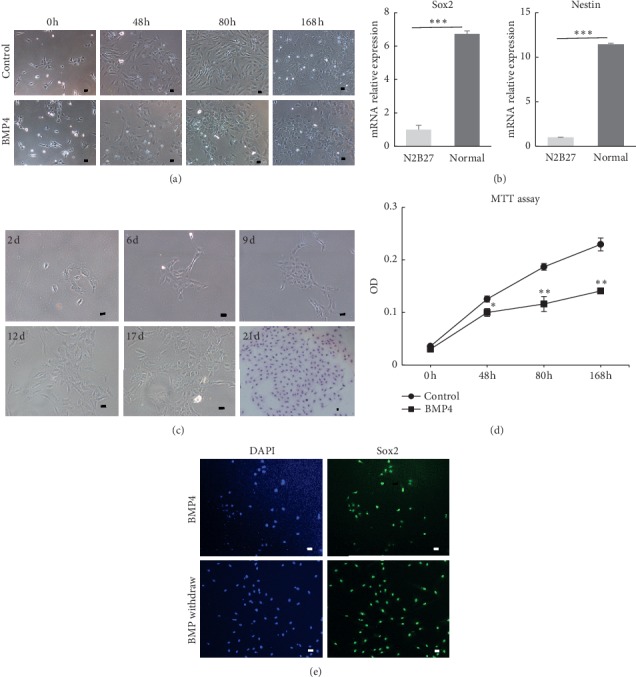
BMP4 inhibited monkey NSC proliferation and maintained Sox2 expression. (a) BMP4 slowed down cell growth during 168h. Cell morphology showed a significant difference in the BMP4 group. Scale bar = 50 *μ*m. (b) Nestin and Sox2 genes detected in monkey NSCs. Normal: medium with growth factor. N2B27: medium without growth factor. ^*∗∗*^*P* < 0.01, ^*∗∗∗*^ *P* < 0.001. Data represent the mean values ± SD. (c) Clone formation in monkey NSCs. 21 d was Giemsa staining. d: culture days. Scale bar = 50 *μ*m. (d) NSC proliferation was detected by MTT assay at 0, 48, 80, and 168h after BMP4 treatment. ^*∗*^*P* < 0.05,^*∗∗*^ *P* < 0.01 compared with control. Data are presented as mean values ± SD. (e) BMP4-treated monkey NSCs were Sox2 positive after BMP4 was withdrawn. Scale bar = 50 *μ*m.

Monkey NSCs were derived from the hippocampus and temporal cortex [18]. Monkey NSCs with BMP4 treatment exhibited a multipolar different morphology compared with control (Figure 1(a)). Meanwhile, Sox2 and Nestin were expressed at high levels in monkey NSCs cultured in the presence of EGF, FGF2, and heparin compared with cells cultured in the control medium (Figure 1(b)). We initially observed the ability of single NSCs to form clones as a method to confirm the features of monkey NSCs (Figure 1(c)).

As shown in our previous study, BMP4 regulates the proliferation of monkey NSCs [18]. We performed a time-course experiment to better understand the function of BMP4. NSCs derived from the adult monkey brain showed some differences from human NSCs, which we isolated from human embryonic stem cells [5]. In the presence of FGF2, monkey NSCs exhibited active proliferation, while cell proliferation slowed down in the presence of BMP4 and FGF2 during 168 h (Figure 1(a)). After a 72 h treatment, BMP4 exerted a substantial effect on the number and morphology of NSCs. Cells were flattened with a larger area after the BMP4 treatment. BMP4 reduced the growth rate of NSCs. Moreover, the cell number was significantly decreased after BMP4 stimulation for 48 h (data not shown). Consistent with this, the results of the MTT assay also displayed similar changes (Figure 1(d)).

In addition to these effects of regulating NSC self-renewal, survival, and differentiation [19], BMP4 is known to induce cell cycle exit and quiescence [20]. We further determined whether the BMP4-induced inhibition of proliferation contributed to the quiescence of monkey NSCs. When BMP4 was withdrawn, NSCs began to proliferate and exhibited a similar morphology to the control group after 7 days. Most strikingly, upon 3 days after BMP4 was withdrawn, NSCs maintained Sox2 expression (Figure 1(e)). The results showed that BMP4 had the ability to promote NSCs into a quiescent state. Moreover, NSCs still displayed stem cell features, like Sox2-positive.

### 3.2. BMP4-Treated NSCs Expressed the Functional BMP Signaling Machinery

**Figure 2:**
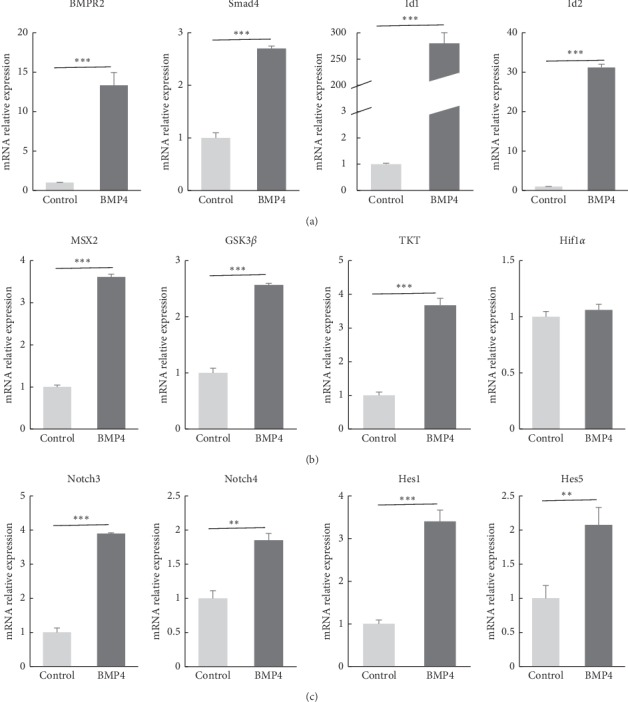
Monkey NSCs expressed functional BMP signaling machinery after BMP4 treatment. (a) BMP signaling pathway relative genes were detected in monkey NSCs. BMPR2, Smad4, Id1, and Id2 were upregulated in BMP4-treated cells compared with control. Data represent the mean ± SD. (b) BMP target genes MSX2, GSK3*β*, TKT, and Hif1*α* were analyzed after BMP4 treatment in monkey NSCs. Expression levels of these genes were significantly higher after treatment with BMP4 except Hif1*α*. Hif1*α* showed no response to BMP. Data represent the mean values ± SD. (c) Genes of the Notch pathway were tested such as Notch1, Notch2, Notch3, Notch4, Hes1, and Hes5. BMP4 had strongly promoted the expression level of Notch3, Notch 4, Hes1, and Hes5 in monkey NSCs. Data represent the mean ± SD. ^*∗∗*^*P* < 0.01,^*∗∗∗*^*P* < 0.001.

To identify possible signaling pathways contributing to the function of BMP4, we assessed the “canonical” BMP-Smad signaling pathways and examined the expression of downstream genes of BMP-Smad signaling, like BMP receptor 2 (BMPR2), Smad4, an inhibitor of DNA binding 1 (Id1), and Id2 [11, 21]. Notably, NSCs treated with BMP4 expressed BMPR2, Smad4, Id1, and Id2 at higher levels (Figure 2(a)).

In addition, the expression of three BMP-targeted genes, muscle segment homeobox gene 2 (MSX2) [22, 23], transketolase (TKT), and GSK3*β*, appeared to be induced to a greater extent by BMP4 in the presence of FGF2. In contrast, hypoxia-inducible factor-1*α* (Hif1*α*) expression response to BMP4 was not altered (Figure 2(b)). These genes are candidate effectors of self-renewal; TKT is involved in cell proliferation [24] and GSK3*β* inhibits Wnt signaling, which is required for proper NSCs self-renewal and differentiation [4, 10].

Although Notch signaling is very important in the quiescence, maintenance, and astrocytic differentiation of embryonic and adult NSCs [8, 25], little is known about the interaction of the Notch signaling pathway with BMP signaling pathways in monkey NSCs. Here, we also investigated the possible role of Notch signaling in BMP4-mediated alterations in monkey NSCs. The addition of BMP4 to monkey NSCs dramatically increased the transcription of the Notch3, Notch4, Hes1, and Hes5 genes (Notch-responsive genes) (Figure 2(c)). The data presented here provide evidence that BMP partially controlled monkey NSC proliferation through Notch signaling pathways.

### 3.3. The Growth of NSCs Arrested in Response to BMP4/LIF or RA/Forskolin Treatment

**Figure 3:**
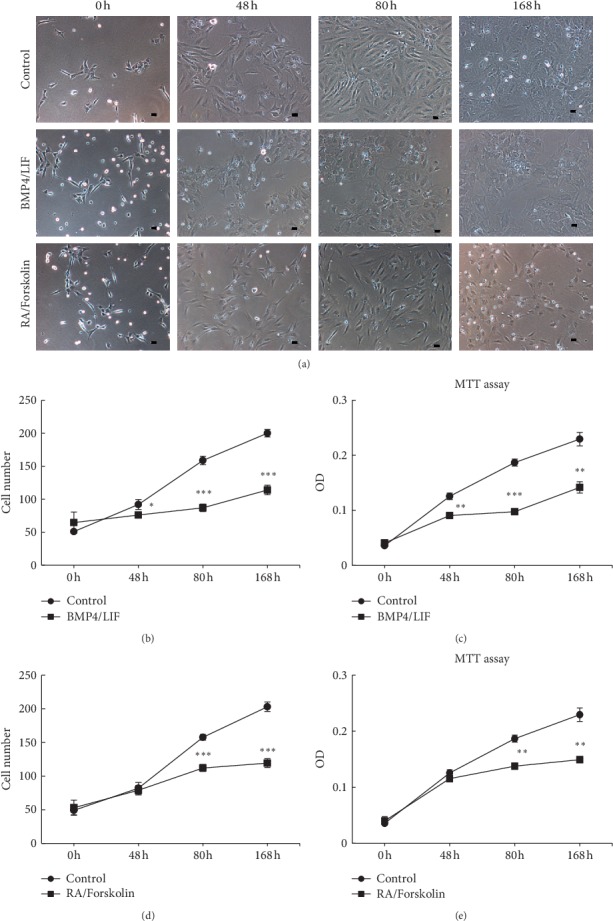
NSC growth slowed down after BMP4/LIF or RA/forskolin treatment. (a) Bright-field images showed that cell growth slowed down after BMP4/LIF or RA/forskolin treatment. Scale bar = 50 *μ*m. (b) and (d) Growth curve showed that NSCs decreased under BMP4/LIF or RA/Forskolin. Cell number was assessed in 6 random fields of each well (3 wells/group). (c) and (e) MTT assay showed BMP4/LIF or RA/forskolin limited cell proliferation. ^*∗*^*P* < 0.05,^*∗∗*^*P* < 0.01,^*∗∗∗*^*P* < 0.001 compared with control. Data represent the mean values ± SD.

To investigate the effects of BMP/LIF or RA/forskolin on monkey NSCs, we observed the changes in cell morphology during the treatment of these cytokines. In the control group, monkey NSCs exhibited an undifferentiated morphology. However, many significant differences were observed when NSCs were treated with BMP/LIF for various times, and many cells had a morphology resembling glial-like cells in BMP4/LIF-treated cultures (Figure 3(a)). After treatment during 168h, RA/forskolin induced NSCs to differentiate and display typical neural phenotypes, such as neuron-like cell bodies and long processes (Figure 3(a)).

It has been shown that BMP4 inhibited cell growth in our study. Others have reported that LIF can promote cell self-renewal, survival, and differentiation in CNS [26]. To assess in depth the growth changes of monkey NSCs in response to BMP4/LIF, we next calculated cell numbers at four time points. When cultured with BMP4/LIF, an initial decrease in cell number was observed beginning at 48 h and the proliferation decreased significantly at 168 h (Figure 3(b)). Regarding cell growth, we also observed a progressive decrease of OD during the 168 h MTT experiments, which reflected the percentage of proliferating cells (Figure 3(c)). Together, these data showed a significant decline of the proliferation of NSCs after BMP/LIF treatment.

RA signaling was shown to promote survival and proliferation and regulate the choice between glial and neuronal fates in adult neural precursor cells [4, 27]. We next investigated the response of NSCs to RA/Forskolin. After 7 days of RA/forskolin treatment, the process of cell division gradually slowed (Figure 3(d)), and many cells exhibited neuron-like cell bodies and long processes (Figure 3(a)). In addition, MTT assay showed that cells grew more slowly in RA/forskolin than cells in the control group during 168 h (Figure 3(e)). Based on these results, combinations of the cytokines BMP4/LIF and RA/forskolin induced growth arrest.

### 3.4. BMP4/LIF or RA/Forskolin Treatment Suppressed the Proliferation of Monkey NSCs

**Figure 4:**
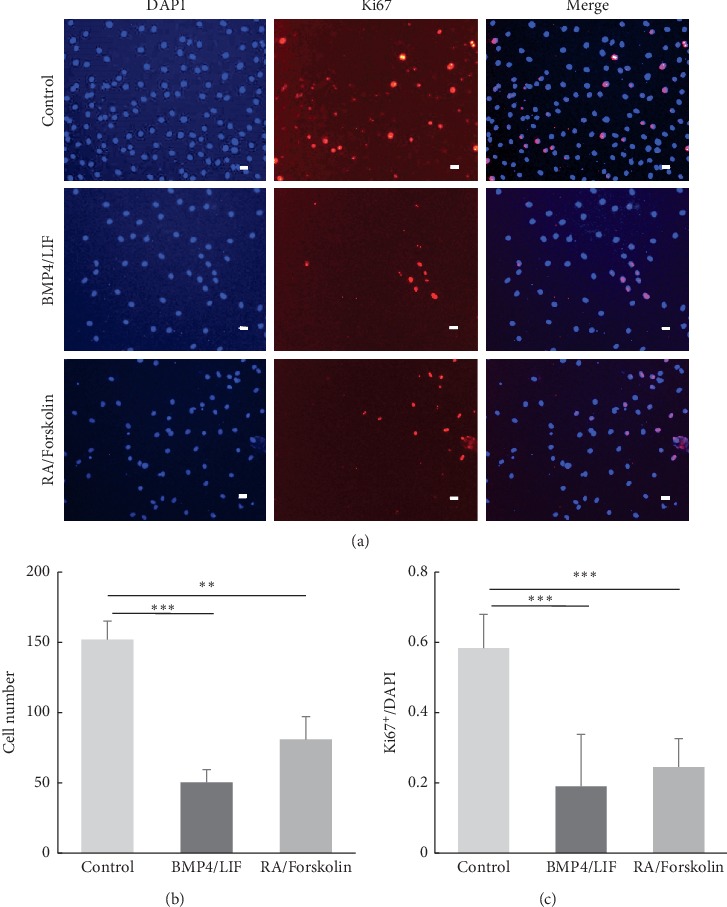
BMP4/LIF or RA/forskolin suppressed cell proliferation. (a) Both BMP4/LIF and RA/forskolin resulted in a significant decrease in Ki67 positive cells. (b) Cell amount presented that BMP4/LIF and RA/forskolin induced proliferation inhibition. Cell number was assessed in 6 random fields of each well (3 wells/group). (c) Percentage of Ki67 positive cells was also strikingly down after BMP4/LIF and RA/forskolin treatment. Data represent the mean ± SD. ^*∗∗*^*P* < 0.01,^*∗∗∗*^*P* < 0.001 were considered statistically significant.

Next, we performed immunofluorescence staining for Ki67, a marker of proliferation, in monkey NSCs to confirm whether growth arrest was attributed to NSC proliferation. More than 24% of cells were labelled with Ki67 (Figures 4(a) and 4(c)). The total cell number decreased after treatment with BMP4/LIF (Figure 4(b)).

Coincident with growth arrest, immunostaining indicated the complete suppression of Ki67 expression by RA/forskolin treatment (Figure 4(a)). RA/forskolin treatment decreased the number of cells at 7 days (Figure 4(b)). The proportion of Ki67-positive cells also decreased from 58.4% to 24.5% (Figure 4(c)). Altogether, BMP4/LIF or RA/forskolin inhibited NSC proliferation.

### 3.5. BMP4/LIF or RA/Forskolin Treatment Promoted the Differentiation of Monkey NSCs

**Figure 5:**
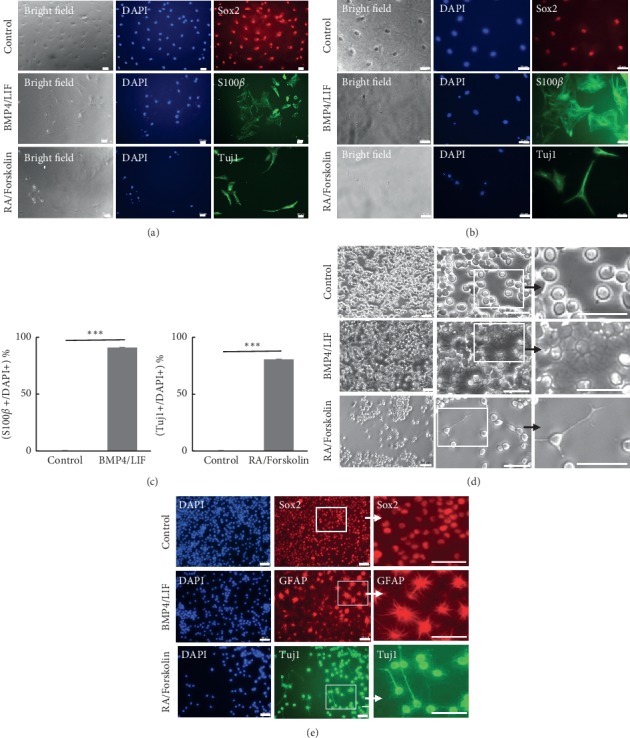
NSCs could differentiate into astrocyte-like and neuron-like after BMP4/LIF or RA/forskolin treatment. (a) BMP4/LIF or RA/forskolin induced monkey NSC differentiation. BMP4/LIF promoted monkey NSCs to astrocyte-like cells and RA/forskolin encouraged monkey NSCs to neuron-like cells. (b) Higher magnification of (a). (c) Ratio of positive cells in monkey NSCs induced by BMP4/LIF or RA/Forskolin. Cell number was assessed in 6 random fields of each well (3 wells/group). Data represent the mean ± SD. ^*∗∗∗*^*P* < 0.001 was considered statistically significant. (d) Cell morphology showed that BMP4/LIF or RA/forskolin induced the differentiation of HCN cells. The right column is the higher magnification of the area with a white frame in the middle. (e) BMP4/LIF also promoted rat hippocampal neural stem cells to astrocyte-like cells and RA/forskolin encouraged rat hippocampal neural stem cells to neuron-like cells. Neural stem cells were stained with Sox2, GFAP, and Tuj1. The right column is the higher magnification of the area with a white frame in the middle. All scale bars = 50 *μ*m.

We assumed that proliferative suppression of monkey NSCs may be due to the differentiation of NSCs, as evidenced by glia or neuron-like morphology after the treatment of these factors. To further validate this result, we also analyzed the lineage-specific differentiation by immunofluorescence staining. In the normal culture, monkey NSCs expressed the NSC-specific related Sox2. After treatment with BMP4/LIF, a large percentage of NSCs (91% ± 7.77) differentiated into S100*β*-positive astrocytes and extend many dendrites that formed an interconnected network (Figures 5(a)–5(c)). In addition, more than 80% of Tuj1-positive cells with the extension of a long axon appeared after RA/forskolin exposure, suggesting the stimulation of neural differentiation (Figures 5(a)–5(c)). Thus, the results showed that BMP4/LIF or RA/forskolin triggers monkey NSCs to differentiate into glia or neuron cell lineages.

Consistent with these results above, we used BMP4/LIF or RA/forskolin to treat HCN cells. Then, we found that HCN cells could differentiate into glia or neurons. Neuron-like differentiation could be achieved by RA/Forskolin, which induced cells to extend a long axon. BMP4/LIF induced cells to extend many dendrites and interlace into a network (Figure 5(d)). In the presence of basic FGF2, HCN expressed NSC-specific marker Sox2 (Figure 5(e)). After BMP4/LIF or RA/Forskolin, these cells differentiated into glia or neurons like cells expressing astrocyte-related gene GFAP and neuron-related gene Tuj1 (Figure 5(e)).

### 3.6. Notch and Jak/Stat Signaling Pathways Promoted Monkey NSC Differentiation

**Figure 6:**
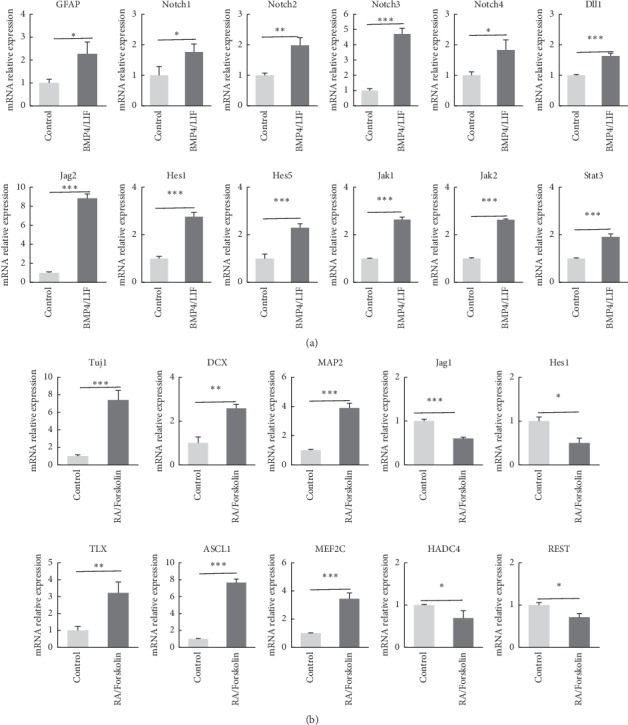
BMP4/LIF and RA/forskolin may promote monkey NSC differentiation by inducing the Notch signaling pathway. (a) BMP4/LIF induced the expression of Notch signaling pathway genes and GFAP. BMP4/LIF increases the expression of genes downstream of the Notch pathway and Jak/Stat pathway such as Notch1-4, Dll1, Jag2, Hes1, Hes5, Jak1, Jak2, and Stat3. (b) Neural differentiation-related genes were analyzed, such as Tuj1, DCX, and MAP2, Notch pathway genes Jag1 and Hes1, proneural genes TLX, ASCL1, and Mef2c, and differentiation repressor genes HDAC4 and REST. High expression levels of Tuj1, MAP2, and DCX appeared after RA and forskolin treatment. RA/forskolin reduced the expression levels of Jag1 and Hes1. Expression levels of TLX, ASCL1, and MEF2C were significantly increased, whereas HDAC4 and REST depressed during neuronal differentiation in monkey NSCs. Data represent the mean ± SD. ^*∗*^*P* < 0.05,^*∗∗*^*P* < 0.01,  and ^*∗∗∗*^*P* < 0.001 were considered statistically significant.

The Notch signaling pathway is known to contribute to the choice of adult neural precursor cells between glial and neuronal lineages [28]. We investigated the effects of BMP4/LIF on the expression of genes downstream of Notch signaling in NSCs and assessed whether the observed effects of these cytokines depended on Notch signaling. We analyzed the expression of Notch1, Notch 2, Notch 3, Notch 4, and the Notch target genes Hes1, Hes5, Jag2 (Jagged2), and delta-like canonical Notch ligand 1 (Dll1). BMP4/LIF induced a small increase in Notch1, Notch2, Notch4, and Dll1 expressions and a 3-fold increase in Hes1 and Notch3 expressions (Figure 6(a)). Furthermore, Jag2 expression was substantially increased by BMP4/LIF (Supplementary Figure S1(a)). BMP4/LIF did not alter Ngn1 levels (Supplementary Figure S1(b)). The expression of most Notch target genes increased, suggesting that the Notch pathway was activated in monkey NSCs treated with BMP4/LIF.

Since LIF has been shown to alter NSC self-renewal, division, and differentiation, all of which are likely mediated by the Jak/Stat pathway [9, 29], we examined the expression of the Jak1, Jak2, and Stat3 genes after the BMP4/LIF treatment. The quantitative analysis confirmed increases in Jak1, Jak2, and Stat3 expressions induced by the BMP4/LIF treatment (Figure 6(a)).

We analyzed the effects of RA/forskolin on monkey NSCs and detected transcriptional responses to examine whether neuronal differentiation induced by RA/forskolin was associated with the expression of neural precursor-related genes. The expression of tubulin beta 3 class III (TUBB3, Tuj1), microtubule-associated protein 2 (MAP2), and doublecortin (DCX) increased after RA/forskolin exposure, suggesting that neuronal differentiation occurred (Figure 6(b)).

Consistent with the activation of neural precursor-related genes, the expressions of the proneural genes homologue of the Drosophila tailless gene (TLX), achaete-scute family bHLH transcription factor 1 (ASCL1 and Mash1), and myocyte enhancer factor 2C (MEF2C) were significantly increased, whereas the expression of transcriptional repressors of neuronal differentiation such as histone deacetylase 4 (HDAC4) and repressor element-1 silencing transcription factor (REST) decreased during the neuronal differentiation of monkey NSCs (Figure 6(b)).

Interestingly, RA/forskolin downregulated the expression of the Notch pathway targets Hes1 and Jag1 in monkey NSCs during the neural differentiation phase (Figure 6(b)). Thus, the Notch pathway was also involved in the differentiation induced by RA/Forskolin.

## 4. Discussion

We obtained NSCs from the adult monkey brain in our previous study [18]. In the present study, we performed additional detailed studies of monkey NSCs to determine the signaling mechanism by which BMP4/LIF induced astrocyte differentiation and RA/forskolin induced neural differentiation. We initially characterized clone formation, the expression of marker genes Nestin and Sox2, and the cell growth rate. Then, cell growth inhibition was observed and the expressions of most BMP signaling-related genes (BMPR2, Smad4, Id1, Id2, MSX2, and TKT) were upregulated in monkey NSCs treated with BMP4. However, monkey NSCs still maintain Sox2-positive, not to differentiation, following treatment with BMP4. This suggested that BMP might have triggered the canonical Smad pathway and the expression of downstream target genes to induce monkey NSC quiescence.

Moreover, our results showed a gene that inhibits Wnt signaling (GSK3*β*) and Notch signaling genes (Notch3, Notch4, Hes1, and Hes5) were upregulated in monkey NSCs treated with BMP4, except Hif1*α*. Hif1*α* expression did not change in response to BMP4, potentially due to the use of normoxic culture conditions. These results implied that the influence of BMP4 in monkey NSCs was achieved by the interaction of multiple signaling pathways like BMP, Wnt, and Notch signaling pathways.

By tracking the changes in monkey NSCs, we observed a deceleration of cell proliferation after BMP4/LIF and RA/forskolin treatments. A corresponding reduction in the percentage of Ki67-positive cells was also observed. Furthermore, BMP4/LIF triggered the glial differentiation of monkey NSCs, resulting in cell morphology changes and S100*β* expression, consistent with the functions of these factors in HCN cells. Exposure to RA/forskolin elicited the neuronal differentiation of monkey NSCs, as identified by the expression of neural differentiation-related genes and proneural genes, low levels of differentiation repressor genes, and a decrease in cell growth. Based on these findings, BMP/LIF or RA/forskolin might be sufficient as a differentiation therapy targeting transplanted stem cells.

Previous studies confirmed that the Notch signaling pathway affected a wide range of cellular processes in NSCs, including cell differentiation [8, 9, 28]. Therefore, we explored whether Notch signaling pathway was involved in the changes of monkey NSCs induced by BMP4/LIF or RA/forskolin treatments. The expressions of most Notch family genes, such as Notch1, Notch2, Notch3, Notch4, and Notch target gene Dll1, were upregulated by BMP4/LIF treatment. In contrast, the expression of the Notch pathway genes Jag1 and Hes1 decreased in response to RA/forskolin exposure. Our research firstly showed that BMP4/LIF and RA/forskolin promoted monkey NSC differentiation at least in part through the Notch signaling pathway.

The glial differentiation of BMP4/LIF-treated NSCs involved different pathways, as LIF activates Stat3 to maintain the undifferentiated state of mouse ESCs [30]. A series of studies have revealed that astrocyte-inducing cytokines function as negative regulators of bHLH factors to inhibit neurogenesis [31]. BMP exerts an antineurogenic effect on neuroepithelial cells by inducing the expression of antineurogenic bHLH proteins [31, 32]. In addition, we explored the response genes and related signaling pathways that were activated in monkey NSCs treated with BMP4/LIF. The expression of Hes1, Hes5, Jak1, Jak2, and Stat3 increased in monkey NSCs after BMP4/LIF treatment. This finding supported the hypothesis that BMP4/LIF promoted the differentiation to astrocytes via bHLH regulators and the Jak/Stat pathway.

What are the key roles of BMP4, BMP4/LIF, and RA/forskolin treatment in controlling the final fates of these monkey NSCs? What are the functions of these factors in the NSC transplantation application? In a future study, we will try to answer these questions and explore the key intrinsic signaling pathways among the large numbers of corresponding genes activated by these factors. The identification of key signaling pathways may improve our understanding of cell transplantation and provide some useful theories for cell therapy.

## 5. Conclusions

We performed a detailed analysis of monkey NSCs after treatment with cytokines like BMP4, BMP4/LIF, and RA/Forskolin. Based on our results, BMP4 might induce monkey NSC quiescence and affect cell proliferation through the Smad signaling pathway. Meanwhile, BMP4/LIF and RA/forskolin might promote the differentiation of monkey NSCs by regulating Notch signaling. Thus, monkey NSCs might initially serve as a potential source of transplant cells to treat neurological degenerative diseases. The identification of signaling mechanisms activated by these cytokines will provide theoretical evidence for NSC therapy in regenerative medicine.

## Data Availability

The data used to support the findings of this study are included within the article and the supplementary information.

## Supplementary Materials

Supplementary Materials Figure S1: the expression of Jag2 (a) and Ngn1 (b) in monkey NSCs treated with BMP4/LIF. ^*∗∗∗*^*P* < 0.001. Table S1: the qPCR primer sequences for various genes.
