# Supplementary material for: Cytokines Induce Monkey Neural Stem Cell Differentiation through Notch Signaling
Source: Biomed Res Int. 2020 May 13;2020:1308526. doi: 10.1155/2020/1308526 (PMC7244951; doi:10.1155/2020/1308526)
Supplement: Supplementary Materials — Figure S1: the expression of Jag2 (a) and Ngn1 (b) in monkey NSCs treated with BMP4/LIF. ∗∗∗P < 0.001. Table S1: the qPCR primer sequences for various genes. [file 1308526.f1.zip › Supplementary materials Table. 1.docx]

**Supplementary Materials**

Supplementary Table. S1 *The qPCR primer sequences for various genes.*

| Gene | Sense primer | Anti-sense primer |
| --- | --- | --- |
| Sox2 | 5’-GCGGAAAACCAAGACGCTCA-3’ | 5′-ATGCGCGTAACTGTCCATGC-3′ |
| Nestin | 5’-TGGCAAGAGGCCGGTACA-3’ | 5′-CCGTATTTGTCCTTCACCTTC-3′ |
| BMPR2 | 5’-AGCAGATCCTGGGCCATCAA-3’ | 5′-TCACCTATCTGTATACTGCTGCCA-3′ |
| Smad4 | 5′-GAGAGCAAGGTTGCACATAGGC-3′ | 5′-TGGTCGCTAAGGCACCTGAC-3′ |
| Id1 | 5′-GGACCTGCAGTTGGAGCTGA-3′ | 5′-GAACGCATGCCGCCTCG-3′ |
| Id2 | 5′-GACCACCCTCAACACGGACA-3′ | 5′-TGAACACCGCTTATTCAGCCAC-3′ |
| MSX2 | 5′-TGAAGCCCTTCGAGACCGC-3′ | 5′-GTAGGGCTCATGTGTCTTGGC-3′ |
| GSK3β | 5′-TGTTCATGATTTGGTGTGGCTGT-3′ | 5′-CATGTGTTGGTTACCTGGGAGG-3′ |
| TKT | 5′- GAACATGGTGAGCATCGCGG -3′ | 5′- ATCTGGTCAAAGGCCCGTGT-3′ |
| Hif1α | 5′-CAAAACACACAGCGAAGC-3′ | 5′-TCAACCCAGACATATCCACC-3′ |
| GAPDH | 5′-CCTGCACCACCAACTGCTTA-3′ | 5′-CATGAGTCCTTCCACGATACCA-3′ |
| Notch1 | 5′- GGACTCAGCAGCACCTGGAT -3′ | 5′- GGGGGTGAAGCCGTCAGG -3′ |
| Notch2 | 5′- AGGAGGCGACCGAGAAGATG -3′ | 5′- TCTCGACACTGCAAGGCACG -3′ |
| Notch3 | 5′- ACCAACCTGGCAGGGAGTTT -3′ | 5′- TTCAGGCATGGGTTGGGGTC -3′ |
| Notch4 | 5′- TCTCGCTTGCTAGCTGGGAC -3′ | 5′- ATCTACGGACGAGCTCGGGA -3′ |
| Hes1 | 5′- CAGATGACGGCTGCGCTGAG -3′ | 5′- GCGCACCTCGGTATTAACGC -3′ |
| Hes5 | 5′- AGGGACCCTCCCCTAAGGAC -3′ | 5′- AACCCTGGTACCTGGCCAAC -3′ |
| GFAP | 5′- GCAGATCCGAGAAACCAGCC -3′ | 5′- GGGTCCTGCCTCACATCACA -3′ |
| Dll1 | 5′- GGCTGGAAAGGGCCCTACTG -3′ | 5′- CCCACTCTGCACTTGCATTCC -3′ |
| Jag1 | 5′- CCAGATGGGGCCAAATGGGA -3′ | 5′- GAGGGCCACACCAGACCTTT -3′ |
| Jag2 | 5′- CTGCCTTGCTACAACGGTGG -3′ | 5′- GTTGATGCGGCAGTCAGGTC -3′ |
| Jak1 | 5′- ATTGCGGCAAGAAGGAAGCG-3′ | 5′- TGCCCCTGCTCAGACTTCTC -3′ |
| Jak2 | 5′- TCGCCCCATCTGTGTAACCG -3′ | 5′- AATATTTGCCGTCGCGGGAG -3′ |
| Stat3 | 5′- TGTGTGACACCATTCATTGATGC -3′ | 5′- CCTCACATGGGGGAGGTAGC -3′ |
| TuJ1 | 5′- GATGAGCATGGCATCGACCC -3′ | 5′- CACGTACTTGTGAGAGGAGGC-3′ |
| DCX | 5′- TTCGCAGCTTTGACGCCTTG -3′ | 5′- AGCTTTCCCCTTCCTCCAGT -3′ |
| MAP2 | 5′- GGACACTCAAGATGATGATAGGAGC -3′ | 5′- GGAAATTCTGCCTCTCCCGGT -3′ |
| TLX | 5′- GGCTGTATCTGGCATGAACGG -3′ | 5′- ATCGAGCCACCACCTCTTGT -3′ |
| ASCL1 | 5′- TGAAAGCAGGGTGATCGCATAA -3′ | 5′- GGCATGCCTCGCTTGGTTG-3′ |
| MEF2C | 5′- GGATGAACGTAACAGACAGGTAACA-3′ | 5′- ACAGCACGCTCAGCTCGTAA -3′ |
| HDAC4 | 5′- CTCACAGGGAACACGGGACA -3′ | 5′- CAACTGAATTCGGCAGCTCGG -3′ |
| REST | 5′- AGCGAGTATCACTGGAGGAAACA-3′ | 5′- TATGGGCGTTCTCCTGTATGAGT -3′ |
| Ngn1 | 5′- GCCCCTGAAGACGAGGTGAA-3′ | 5′-TCAGCCGGCTCAAACCGAAT-3′ |
