# Supplementary material for: Cytokines Induce Monkey Neural Stem Cell Differentiation through Notch Signaling
Source: Biomed Res Int. 2020 May 13;2020:1308526. doi: 10.1155/2020/1308526 (PMC7244951; doi:10.1155/2020/1308526)
Supplement: Supplementary Materials — Figure S1: the expression of Jag2 (a) and Ngn1 (b) in monkey NSCs treated with BMP4/LIF. ∗∗∗P < 0.001. Table S1: the qPCR primer sequences for various genes. [file 1308526.f1.zip › Supplementary fig s1.pptx]

## Slide 1
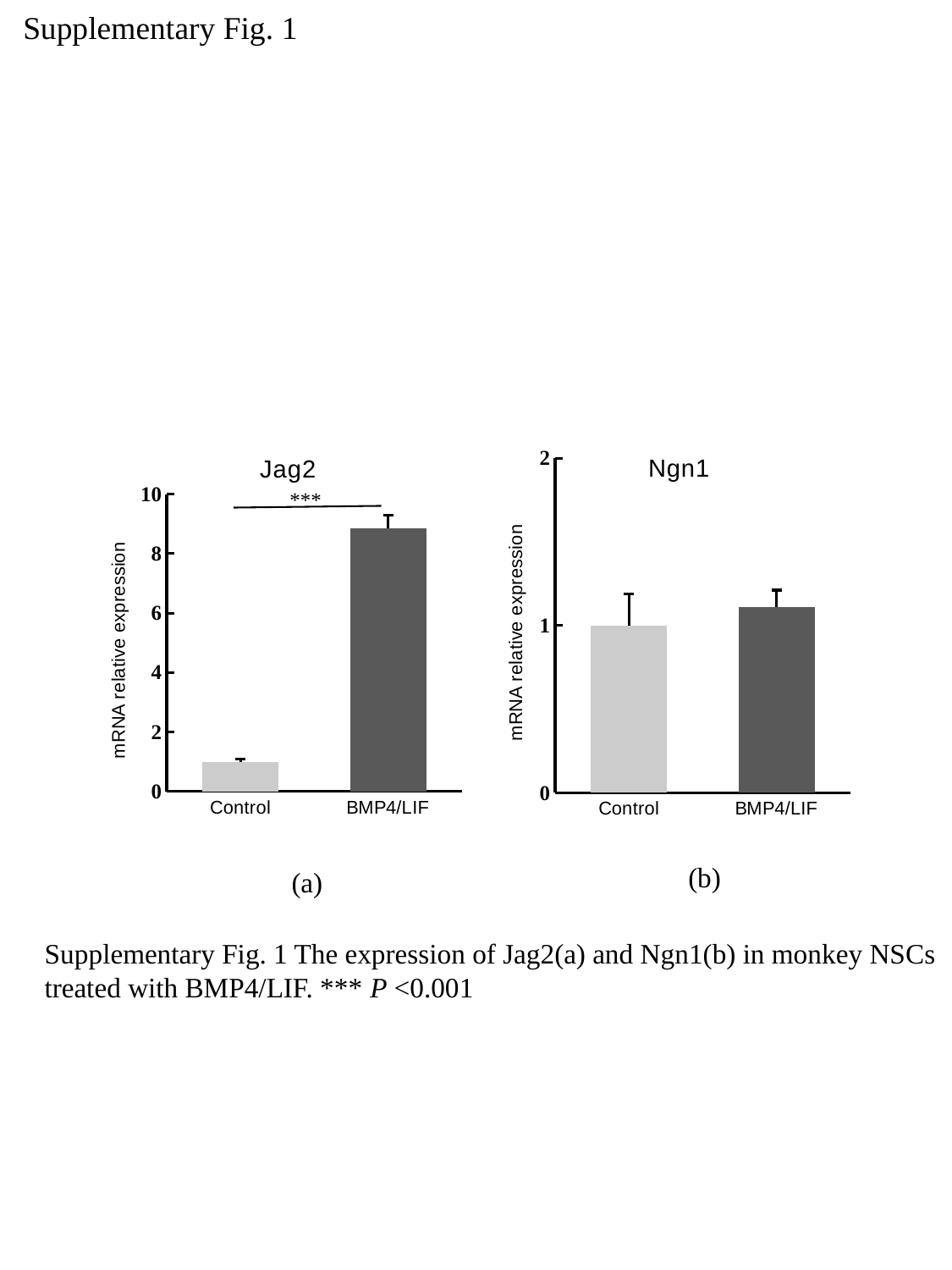

Supplementary Fig. 1
### Chart: Jag2
| Category | |
|---|---|
| Control | 1.0 |
| BMP4/LIF | 8.834229 |
### Chart: Ngn1
| Category | |
|---|---|
| Control | 1.0 |
| BMP4/LIF | 1.110323 |(b)
(a)
Supplementary Fig. 1 The expression of Jag2(a) and Ngn1(b) in monkey NSCs treated with BMP4/LIF. *** P <0.001
